# Gel Polymer Electrolyte Membranes via Slit-Coating Technology for High-Energy Lithium Batteries

**DOI:** 10.3390/gels12060534

**Published:** 2026-06-14

**Authors:** Pengzhen Chen, Xinghua Liang, Te Zheng, Lei Zhang, Jiajia Dong, Yangying Ou, Lingxiao Lan, Jianghua Wei

**Affiliations:** Guangxi Key Laboratory of Automobile Components and Vehicle Technology, Guangxi University of Science & Technology, Liuzhou 545006, China; 15110761348@163.com (P.C.); lxh304@126.com (X.L.); zzz593756895@163.com (L.Z.); 16650298814@163.com (J.D.); 15697920119@163.com (Y.O.);

**Keywords:** gel electrolyte membranes, slit coating, UV curing process, LATP, LLZTO

## Abstract

Liquid electrolytes in conventional lithium-ion batteries pose safety risks associated with flammability, leakage, and explosion, whereas solid polymer electrolytes are generally limited by insufficient ionic conductivity at ambient temperature, restricting the development of high-energy lithium batteries. To address these issues, flexible poly (vinylidene fluoride-co-hexafluoropropylene) (PVDF-HFP)-based gel polymer electrolyte membranes (GPEs) were prepared via a slit-coating process combined with UV curing. NASICON-type lithium aluminum titanium phosphate (Li_1.3_Al_0.3_Ti_1.7_P_3_O_12_, LATP) and garnet-type tantalum-doped lithium lanthanum zirconate (Li_6.4_La_3_Zr_1.4_Ta_0.6_O_12_, LLZTO) were introduced as inorganic ceramic fillers to improve the ion-transport and interfacial properties of the GPE. Among the investigated samples, the PVDF-HFP-based GPE containing 10 wt% LLZTO exhibited the best overall performance, with an ionic conductivity of 3.40 × 10^−4^ S·cm^−1^ at ambient temperature and a Li^+^ transference number of 0.77. Cyclic voltammetry results showed that the LLZTO-modified electrolyte membrane exhibited sharper and more symmetric redox peaks, higher peak current response, and better curve overlap during repeated cycles, indicating improved electrochemical reversibility and interfacial stability. In addition, LLZTO incorporation enhanced the mechanical strength, broadened the electrochemical stability window, and improved the flame-retardant behavior of the membrane. The LiFePO_4_/GPE/Li cell assembled with the optimized membrane delivered an initial discharge capacity of 160 mAh·g^−1^ at 0.1 C and maintained 80 mAh·g^−1^ at 1 C, demonstrating good rate capability. Moreover, a capacity retention of 96% was maintained after 100 cycles at 0.1 C, confirming excellent cycling stability. Therefore, this work provides an effective strategy for the structural optimization and scalable preparation of high-performance gel polymer electrolyte membranes for lithium battery applications.

## 1. Introduction

Lithium-ion batteries (LIBs) serve as essential energy storage devices and are broadly employed in portable electronics, electric vehicles, and renewable energy applications, promoting higher energy efficiency and improved living standards [1]. Liquid electrolytes in lithium-ion batteries display volatility, flammability, and a tendency to leak, leading to safety concerns [2,3]. Moreover, during the cycling process, they are prone to inducing lithium dendrite growth, which may cause internal short circuits and even thermal runaway, severely restricting the application of batteries under higher energy density and more demanding operating conditions [4,5,6]. With their wide electrochemical window, mechanical robustness, and stable nature, solid electrolyte membranes (SEMs) are well-suited for pairing with lithium metal and high-voltage cathodes, offering a viable route toward safer and higher-energy-density batteries [7,8,9].

Among different SEM systems, gel polymer electrolyte membranes (GPE) are constructed by integrating inorganic ceramic fillers within the polymer matrix. By combining polymer flexibility with the superior ionic transport properties of inorganic ceramics, these GPEs exhibit considerable promise for advanced electrochemical applications [10,11,12]. Among them, poly(vinylidene fluoride-co-hexafluoropropylene) is often selected as the polymer matrix because of its excellent oxidation resistance, good electrochemical stability, and ease of processing [13,14]. To further enhance the ionic conductivity and mechanical properties of GPEs, fast ion-conductive ceramic fillers are usually introduced, such as NASICON-structured lithium aluminum titanium phosphate (Li_1.3_Al_0.3_Ti_1.7_P_3_O_12_, LATP) and garnet-type tantalum-doped lithium lanthanum zirconate (Li_6.4_La_3_Zr_1.4_Ta_0.6_O_12_, LLZTO) [15,16,17]. LATP is characterized by high ionic conductivity and structural stability, while LLZTO shows favorable compatibility with lithium metal and a high Li^+^ transference number [18,19]. Therefore, incorporating these inorganic ceramic fillers into the PVDF-HFP matrix provides a feasible material design strategy for regulating the microstructure and improving the electrochemical performance of GPE.

Furthermore, the engineering application of electrolyte membranes depends not only on material composition but also on the scalability and controllability of the preparation process. In laboratory studies, methods such as knife coating and solution casting are commonly used to prepare electrolyte membranes; however, these methods often suffer from limited thickness uniformity, poor process reproducibility, and difficulty in meeting the requirements of continuous large-area production [20,21]. In contrast, slit coating possesses a highly precise and scalable film preparation process, which demonstrates unique advantages in the preparation of uniform and large-area electrolyte membranes [22,23]. This process can achieve uniform membrane thickness and quantitative coating through precise control of parameters such as slurry flow rate, coating gap, substrate speed, and tension, and it has good process repeatability, which is an important way to promote solid-state batteries from the laboratory to industrialization [24,25,26]. In addition, the integration of slit coating with UV curing further improves the practical applicability of this preparation strategy. UV curing enables rapid in situ polymerization and crosslinking of the coated wet film, which can shorten the fabrication time, improve membrane structural stability, and reduce defects caused by solvent evaporation or film deformation. More importantly, the slit-coating/UV-curing process is compatible with continuous roll-to-roll manufacturing, making it suitable for large-area and scalable production of GPE membranes. However, most current studies focus mainly on single-filler systems and lack systematic comparisons of lithium-ion transport behavior and interfacial characteristics in PVDF-HFP-based GPE containing LATP and LLZTO fillers at different ratios. Meanwhile, reports on membrane preparation and performance evaluation using this process remain relatively limited [27,28].

Previous studies have shown that an appropriate amount of ceramic filler can reduce polymer crystallinity and construct additional Li^+^ migration pathways, thereby improving ionic conductivity [29]. However, excessive filler loading may cause particle aggregation and hinder polymer-chain mobility, leading to increased ion-transport resistance. Therefore, optimizing the filler content is essential for obtaining high-performance GPE. In this study, the slit-coating process was employed using PVDF-HFP polymer as the matrix, and different mass fractions (0, 5, 10, and 15 wt%) of LATP or LLZTO ceramic fillers were introduced to prepare GPEs. The influence of filler type and concentration on the microstructure, thermal and mechanical properties, as well as ion-transport characteristics of the GPEs, including lithium-ion transference behavior, was systematically analyzed. Through a comprehensive comparison of the two filler systems, the electrolyte membrane with the best ionic transport performance was selected and further assembled into LiFePO_4_/GPEs/Li batteries to evaluate its electrochemical cycling performance at room temperature. This study establishes experimental and theoretical foundations for advancing lithium-ion batteries with enhanced safety and energy density.

## 2. Results and Discussion

**Figure 1:**
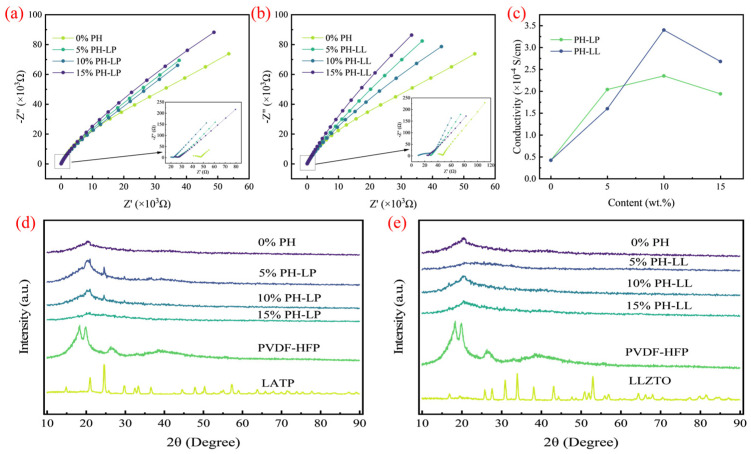
(**a**,**b**) Impedance spectra of SS/GPE/SS symmetric cells at ambient temperature (inset: magnified high-frequency region); (**c**) ionic conductivity as a function of LATP or LLZTO content; (**d**,**e**) XRD profiles of GPE with different formulations.

Ionic conductivity is a critical parameter for evaluating the electrochemical performance of electrolyte membranes, as it directly determines their practical applicability in battery systems [30,31]. Figure 1a,b show the Nyquist plots of PH-LP and PH-LL membranes with different filler contents measured using a stainless-steel/electrolyte membrane/stainless-steel (SS/GPE/SS) blocking-electrode configuration. In this configuration, the resistance obtained from the high-frequency intercept is mainly associated with the bulk resistance or overall ion-transport resistance of the electrolyte membrane, rather than the interfacial resistance of a full cell. Compared with the pristine 0% PH membrane, the introduction of LATP or LLZTO significantly decreases the bulk resistance of the electrolyte membranes, indicating improved ion transport within the polymer matrix. For the LATP-based membranes, the resistance value decreases from 47 Ω for the 0% PH membrane to 25 Ω, 23 Ω, and 27 Ω at LATP loadings of 5, 10, and 15 wt%, respectively. Similarly, for the LLZTO-based membranes, the corresponding resistance values are 25 Ω, 15 Ω, and 30 Ω, respectively. As summarized in Figure 1c, both systems exhibit the highest ionic conductivity at a filler content of 10 wt%, reaching 2.35 × 10^−4^ S·cm^−1^ for 10% PH-LP and 3.40 × 10^−4^ S·cm^−1^ for 10% PH-LL, which are markedly higher than that of the filler-free membrane, 4.20 × 10^−5^ S·cm^−1^. The enhanced ionic conductivity is closely related to the dispersion state and crystal structure of the ceramic fillers. Well-dispersed LATP or LLZTO particles can disturb the ordered packing of PVDF-HFP chains, reduce polymer crystallinity, and increase the amorphous regions, thereby promoting polymer segmental motion and facilitating Li^+^ migration [32]. In addition, the ceramic fillers provide additional Li^+^ migration sites and help construct continuous ion-conduction pathways within the polymer matrix. Compared with LATP, LLZTO has a cubic garnet structure with continuous three-dimensional Li^+^ transport channels, which is more favorable for rapid Li^+^ migration. This structural feature helps reduce the tortuosity of Li^+^ transport pathways and improves ion migration across the polymer/ceramic interface, thereby explaining the pronounced decrease in resistance from 47 Ω for the 0% PH membrane to 15 Ω for the 10% PH-LL membrane. Moreover, the better dispersion and interfacial compatibility of LLZTO in the PVDF-HFP matrix further reduce ion-transport resistance and promote the formation of an efficient ion-conduction network. Therefore, the 10% PH-LL membrane exhibits the highest ionic conductivity among the investigated samples [33,34].

To gain deeper insight into the amorphous features of the GPE, X-ray diffraction (XRD) analysis was performed. Figure 1d,e show the XRD patterns of LATP- and LLZTO-based GPEs with different filler contents. It can be seen that the 0% PH membrane presents broad and diffuse peaks at approximately 2θ = 18° and 20°, indicating that its overall structure is mainly amorphous. After adding LATP or LLZTO, the XRD spectra of the GPEs are still dominated by broad peak characteristics, and no obvious enhancement of the polymer crystalline peaks is observed, indicating that the addition of LATP or LLZTO does not disrupt the amorphous structure of the polymer matrix. Although LATP and LLZTO are crystalline ceramic fillers, their incorporation does not induce obvious crystallization of the PVDF-HFP matrix; instead, the well-dispersed ceramic particles can disturb the regular packing of polymer chains and help maintain favorable amorphous regions for Li^+^ transport. At the same time, the characteristic crystalline peaks of LATP and LLZTO are significantly weakened in the composite membranes, suggesting that the inorganic ceramic fillers are well dispersed and exhibit good interfacial coupling within the polymer matrix. Based on the comprehensive analysis of the XRD and EIS results for GPE with different filler contents, 10 wt% LATP or LLZTO was found to be the optimal filler content, as it could effectively balance ceramic filler dispersion and polymer matrix continuity, thereby facilitating the formation of continuous and stable Li^+^ transport channels and resulting in higher ionic conductivity. In contrast, lower filler loading may be insufficient to construct effective ion-conduction pathways, while excessive filler loading may cause local particle aggregation and increase ion-transport resistance. Therefore, in the subsequent study, GPE containing 10 wt% LATP or LLZTO were selected as the research objects for further investigation of their structural characteristics and electrochemical performance.

**Figure 2:**
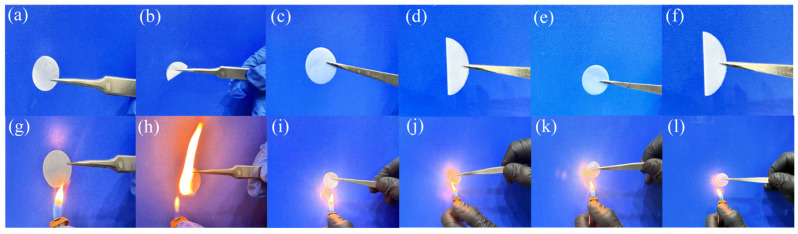
(**a**,**b**) Macroscopic surface images of the 0% PH membrane. (**c**,**d**) Macroscopic surface images of the 10% PH-LP membrane. (**e**,**f**) Macroscopic surface images of the 10% PH-LL membrane. (**g**,**h**) Combustion demonstrations of the 0% PH membrane at 1 s and 5 s, respectively. (**i**,**j**) Combustion demonstrations of the 10% PH-LP membrane at 1 s and 5 s, respectively. (**k**,**l**) Combustion demonstrations of the 10% PH-LL membrane at 1 s and 5 s, respectively.

Figure 2 shows the macroscopic surface morphology and combustion behavior of the 0% PH, 10% PH-LP, and 10% PH-LL membranes, which were used to evaluate the structural integrity and thermal safety of the gel electrolyte membranes. As shown in Figure 2a,b, the 0% PH membrane exhibits a relatively smooth surface and maintains a certain degree of flexibility under bending. After introducing ceramic fillers, the 10% PH-LP membrane (Figure 2c,d) and 10% PH-LL membrane (Figure 2e,f) both show continuous and dense surface morphologies without obvious cracks or delamination, indicating that LATP and LLZTO are well incorporated into the PVDF-HFP matrix. In particular, the 10% PH-LL membrane maintains good structural integrity under bending, suggesting improved flexibility and mechanical stability. The combustion tests were further conducted to compare the flame-retardant behavior of the different membranes. Figure 2g,h show the combustion behavior of the 0% PH membrane at 1 s and 5 s, respectively. The 0% PH membrane exhibits more obvious thermal shrinkage and local deformation under flame exposure, indicating relatively limited thermal stability of the filler-free polymer membrane. In contrast, the 10% PH-LP membrane (Figure 2i,j) and 10% PH-LL membrane (Figure 2k,l) show improved dimensional stability during short-term flame exposure, with no obvious continuous burning behavior. This result demonstrates that the incorporation of LATP or LLZTO ceramic fillers can enhance the flame-retardant stability of the GPEs. Overall, compared with the filler-free 0% PH membrane, the ceramic-filler-modified membranes exhibit better structural integrity and thermal safety, among which the 10% PH-LL membrane shows more favorable comprehensive stability.

**Figure 3:**
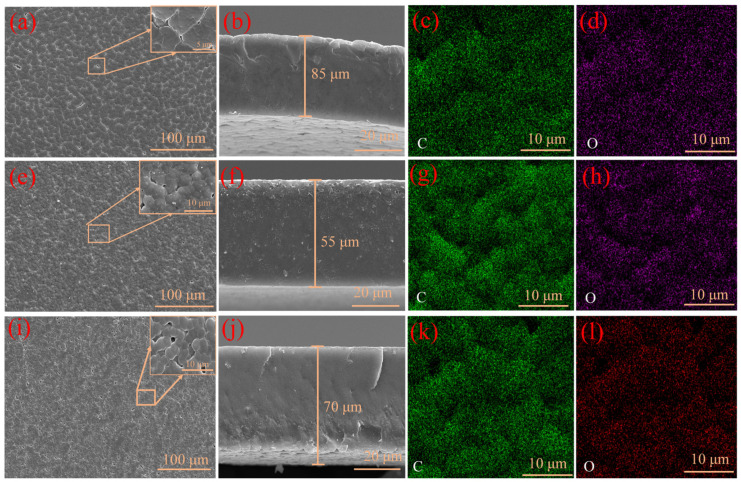
(**a**,**b**) Surface and cross-sectional morphologies of the 0% PH sample; (**c**,**d**) elemental mapping of C and O for the same sample. (**e**,**f**) Surface and cross-sectional features of the 10% PH-LP sample; (**g**,**h**) corresponding C and O elemental distributions. (**i**,**j**) Surface and cross-sectional morphologies of the 10% PH-LL sample; (**k**,**l**) corresponding C and O elemental mappings.

Figure 3a–l show the plan-view and cross-sectional SEM morphologies of the 0% PH, 10% PH-LP, and 10% PH-LL membranes, together with the corresponding EDS elemental mappings of C and O. As shown in Figure 3a–d, the 0% PH membrane exhibits a relatively rough surface and local structural fluctuations. Its cross-section shows a layered morphology with several microcracks, and the membrane thickness is approximately 85 μm. This may be related to the shrinkage of the pure PVDF-HFP-based polymer matrix during solvent evaporation and curing, as well as the absence of inorganic fillers to reinforce the internal structure. After introducing LATP, the 10% PH-LP membrane shows a more compact surface and a denser cross-sectional structure, with a reduced thickness of approximately 55 μm (Figure 3e–h). The decrease in thickness may be attributed to improved slurry dispersion and stronger shrinkage during drying, which leads to a more compact membrane structure. For the 10% PH-LL membrane, a continuous and dense morphology is observed, and its thickness is approximately 70 μm (Figure 3i–l). Compared with LATP, LLZTO may increase the solid content and structural support within the polymer matrix, thereby producing a relatively thicker but more uniform membrane. The thickness difference among the three membranes may influence their ion-transport behavior and electrochemical performance. Generally, a thinner membrane can shorten the Li^+^ migration distance and reduce ohmic resistance, while an excessively thick or structurally defective membrane may increase ion-transport resistance. However, membrane thickness should be considered together with structural integrity, filler dispersion, and interfacial compatibility. Although the 10% PH-LP membrane has the smallest thickness, the 10% PH-LL membrane exhibits a denser morphology, more uniform elemental distribution, and better structural continuity, which are beneficial for constructing stable Li^+^ transport pathways. In addition, the actual membrane thickness was used in the ionic conductivity calculation to avoid errors caused by directly comparing resistance values alone. Since membrane thickness can also affect the areal mass loading and internal resistance of the assembled cells, this factor was considered when discussing the electrochemical performance. Overall, the SEM and EDS results indicate that LLZTO shows better dispersion and interfacial compatibility in the PVDF-HFP matrix, thereby contributing to improved structural integrity and ion-transport properties.

Mechanical properties of polymer electrolytes play a key role in ensuring the safe operation and practical assembly of solid-state lithium-ion batteries [35]. Mechanical failure of the gel electrolyte membrane may be triggered when internal stress surpasses its strength, leading to compromised electrochemical performance [36]. The tensile strength of the 0% PH membrane is 3.06 MPa, while that of the 10% PH-LP and 10% PH-LL membranes increase to 3.52 and 4.72 MPa, respectively, indicating that the introduction of LATP or LLZTO ceramic fillers can enhance the mechanical strength of the PVDF-HFP-based GPEs. The improvement in tensile strength is strongly associated with the dispersion state of the inorganic fillers and their interaction with the polymer matrix. Uniformly dispersed ceramic particles can act as reinforcing sites within the polymer network, restrict polymer-chain slippage, and promote stress transfer under external force, thereby improving the mechanical stability of the membrane [37]. Compared with LATP, LLZTO shows a more pronounced reinforcing effect, which may be attributed to its better dispersion and stronger interfacial compatibility with the PVDF-HFP matrix. The stable garnet crystal structure of LLZTO also provides rigid inorganic support within the flexible polymer matrix, helping to inhibit crack propagation and maintain structural integrity during deformation. Therefore, under the same filler content, the 10% PH-LL membrane exhibits superior mechanical strength and better structural stability, which are beneficial for practical assembly and operation of solid-state lithium batteries [28,38].

**Figure 4:**
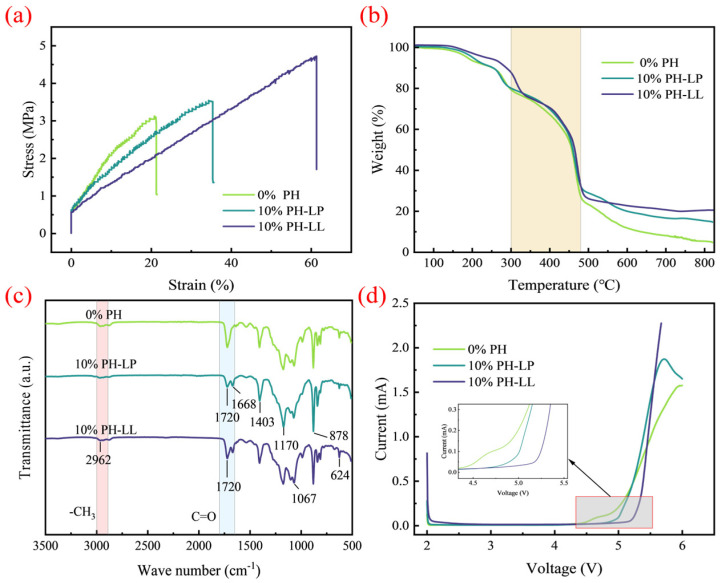
(**a**) Stress–strain curves of the 0% PH, 10% PH-LP, 10% PH-LL membrane. (**b**) TGA curves of the 0% PH, 10% PH-LP, 10% PH-LL membrane. (**c**) FTIR spectra of the 0% PH, 10% PH-LP, 10% PH-LL membrane. (**d**) Electrochemical window of the 0% PH, 10% PH-LP, 10% PH-LL membrane.

The factors affecting the safety of batteries also include the thermal stability of the electrolyte [39,40]. Figure 4b shows the TG profiles of 0% PH, 10% PH-LP, and 10% PH-LL membranes, which were used to evaluate their thermal stability. None of the three membranes showed significant weight loss before approximately 250 °C, indicating that all three membranes have good thermal stability within the actual operating temperature range. As the temperature increased further, the 0% PH membrane rapidly decomposed in the 300–480 °C range, and the residual weight was relatively low, indicating that the pure polymer system is structurally unstable at high temperatures. By contrast, the incorporation of inorganic ceramic fillers noticeably delays the weight-loss process of the GPEs and increases the residual mass at elevated temperatures. At 800 °C, the 10% PH-LL membrane shows a higher residue (20.46%) than the 10% PH-LP (15.26%) and 0% PH (5.24%) membranes. These results indicate that LLZTO enhances the resistance of the membrane to thermal decomposition, thereby improving its overall thermal robustness, which is sufficient to satisfy the operational requirements of lithium-ion batteries.

Figure 4c presents the FTIR spectra of the 0% PH, 10% PH-LP, and 10% PH-LL membranes. All three membranes retain the characteristic absorption bands of PVDF-HFP, indicating that the polymer backbone remains structurally stable during membrane fabrication. The absorption peak at 2962 cm^−1^ is assigned to the stretching vibration of –CH_2_ groups, while the bands in the range of 1170–1067 cm^−1^ are mainly associated with C–F and C–C vibrations in the PVDF-HFP matrix [41]. Compared with the 0% PH membrane, the 10% PH-LP and 10% PH-LL membranes exhibit newly emerged or enhanced absorption bands near 1668 cm^−1^ and 1403 cm^−1^, which can be attributed to the interactions between oxygen-containing groups in LATP/LLZTO and the polymer chain segments [42]. These results suggest that the incorporation of LATP or LLZTO promotes interfacial coupling within the PVDF-HFP network, thereby improving the structural stability of the gel polymer electrolyte membranes.

In addition, linear sweep voltammetry (LSV) was performed to evaluate the electrochemical stability window of the GPE, which plays a key role in determining their practical applicability in energy storage systems [43]. Figure 4d illustrates the LSV responses of the three gel electrolyte membranes: 0% PH, 10% PH-LP, and 10% PH-LL. As the voltage increases, the current begins to gradually rise, indicating the occurrence of oxidation decomposition reactions. For the 0% PH membrane, the current starts to increase at approximately 4.5 V, and a significant oxidation current appears at around 5.1 V, suggesting a relatively narrow electrochemical stability window for the filler-free polymer electrolyte. After introducing LATP, the initial current increase of the 10% PH-LP membrane shifts to approximately 4.9 V, and the oxidation decomposition potential increases to around 5.4 V, indicating that LATP incorporation improves the oxidative resistance of the electrolyte membrane. However, the current rise of the 10% PH-LP membrane still occurs earlier than that of the 10% PH-LL membrane, suggesting relatively lower high-voltage stability. This may be related to the interfacial compatibility between LATP and the PVDF-HFP matrix, as well as possible interfacial side reactions under high voltage. For the 10% PH-LL membrane, the onset voltage further increases to approximately 5.2 V, and the oxidation decomposition potential is delayed to above 5.6 V, showing the widest electrochemical stability window among the three membranes. The improved high-voltage stability of the 10% PH-LL membrane can be attributed to the stable garnet structure of LLZTO and its better interfacial compatibility with the PVDF-HFP matrix, which help suppress electrolyte decomposition at high voltage and maintain a more stable electrode/electrolyte interface. Therefore, although both LATP and LLZTO are ceramic fillers, their different crystal structures and interfacial characteristics lead to different electrochemical stability behaviors. The above results demonstrate that LATP/LLZTO incorporation significantly improves the oxidative resistance of the electrolyte, among which LLZTO is more effective in broadening the electrochemical stability window and is more suitable for high-energy-density lithium battery applications [44,45].

To further highlight the advantages of the LLZTO-modified GPE developed in this work, a comparison with previously reported PVDF-HFP-based polymer electrolytes containing different inorganic fillers is summarized in Table 1. Compared with other ceramic fillers, the 10% PH-LL membrane in this work exhibits a higher Li^+^ transference number and a wider electrochemical stability window, while maintaining relatively high ionic conductivity. These results further confirm that LLZTO is an effective filler for improving Li^+^ transport and electrochemical stability in PVDF-HFP-based GPEs.

**Figure 5:**
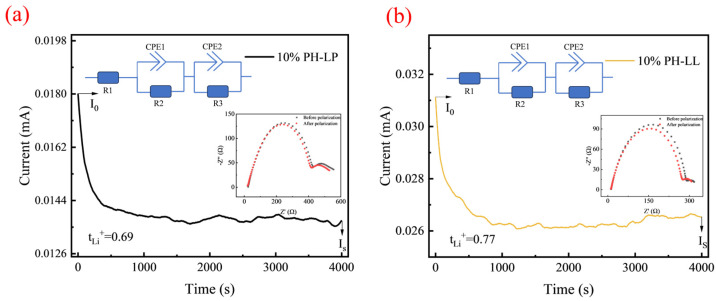
DC polarization curves of Li/GPE/Li symmetric cells based on 10% PH-LP (**a**) and 10% PH-LL (**b**) membranes under a polarization voltage of 0.05 V, together with the impedance spectra before and after polarization. The inset shows the equivalent circuit analysis used to evaluate impedance evolution during polarization.

The Li^+^ transference number (*t_Li_*^+^) is an important parameter for evaluating the ion-transport behavior of electrolyte membranes [48]. In this study, the *t_Li_*^+^ values of the 10% PH-LP and 10% PH-LL membranes were determined using the DC polarization method combined with AC impedance measurements in Li/GPE/Li symmetric cells. Figure 5a,b show the chronoamperometric curves recorded under a constant polarization voltage of 0.05 V, together with the corresponding impedance spectra before and after polarization. The impedance spectra were fitted using the equivalent circuit [*R*1 − (*R*2//*CPE*1) − (*R*3//*CPE*2], as shown in the inset of Figure 5. In this equivalent circuit, *R*1 represents the bulk resistance of the electrolyte membrane, *R*2 is associated with the interfacial resistance between the lithium metal electrode and the electrolyte membrane, and *R*3 corresponds to the charge-transfer resistance at the Li/electrolyte interface. *CPE*1 and *CPE*2 are constant phase elements used to describe the non-ideal capacitive behavior caused by interfacial heterogeneity and surface roughness. The initial current, steady-state current, and impedance fitting parameters before and after polarization used for calculating the Li^+^ transference number are summarized in Table 2.

As shown in Table 2, the *t_Li_*^+^ values of the 10% PH-LP and 10% PH-LL membranes are 0.69 and 0.77, respectively, both of which are higher than those of conventional polymer electrolyte systems. This indicates that the ceramic fillers can effectively promote Li^+^ transport and suppress anion migration. Compared with the 10% PH-LP membrane, the 10% PH-LL membrane exhibits a higher steady-state current and a smaller increase in interfacial resistance after polarization, indicating a more stable Li/electrolyte interface. This improvement can be attributed to the cubic garnet structure of LLZTO, which provides continuous Li^+^ migration pathways and contributes to the formation of a stable, low-impedance ion-conduction network within the composite electrolyte membrane. These findings suggest that LLZTO limits anion migration while enhancing Li^+^ participation in charge transfer, promoting the development of interconnected and low-impedance conduction pathways and resulting in improved ion transport properties [49,50].

**Figure 6:**
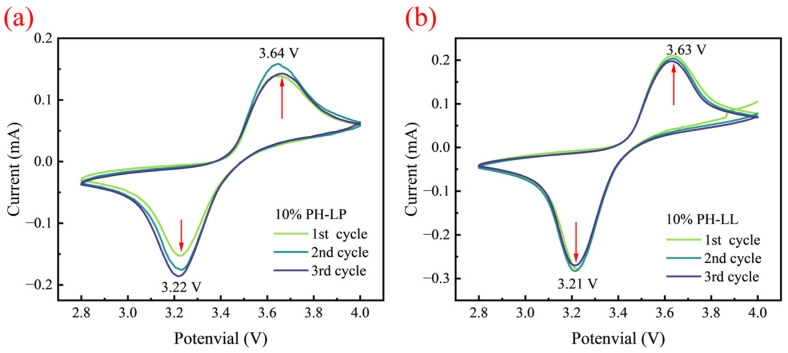
The cyclic voltammetry responses of lithium-ion batteries with 10% PH-LP and 10% PH-LL electrolytes were measured at 0.2 mV·s^−1^. The CV diagram of the 10% PH-LP battery is presented in (**a**), and that of the 10% PH-LL battery is presented in (**b**).

Cyclic voltammetry (CV) is a commonly used electrochemical method for evaluating the reversibility, polarization behavior, and interfacial stability of electrode reactions [51,52]. As shown in Figure 6, both electrolyte systems exhibit a pair of distinct oxidation and reduction peaks, corresponding to the reversible Fe^2+^/Fe^3+^ redox reaction of the LiFePO_4_ cathode, indicating good electrochemical activity of the assembled cells. For the cell based on the 10% PH-LP membrane, the oxidation and reduction peaks are located at 3.64 and 3.22 V, respectively, with a peak separation of approximately 0.42 V. Similarly, for the cell based on the 10% PH-LL membrane, the oxidation and reduction peaks appear at 3.63 and 3.21 V, respectively, also giving a peak separation of approximately 0.42 V. Therefore, the peak separation values of the two cells are comparable, suggesting that both electrolyte membranes enable relatively reversible electrochemical reactions. Although the peak separation of the 10% PH-LL-based cell is not smaller than that of the 10% PH-LP-based cell, the 10% PH-LL cell exhibits a sharper and more symmetric redox peak profile, higher peak current response, and better overlap of the CV curves during the first three cycles. These features indicate improved Li^+^ transport kinetics, lower polarization tendency, and better interfacial stability during cycling. In contrast, the 10% PH-LP cell shows a slight decrease in peak current and a minor peak shift with repeated cycling, suggesting relatively weaker interfacial stability. The improved electrochemical behavior of the 10% PH-LL membrane can be attributed to the cubic garnet structure of LLZTO, which facilitates the formation of continuous Li^+^ migration pathways in the polymer matrix and enhances electrode–electrolyte compatibility. Accordingly, the 10% PH-LL membrane demonstrates better electrochemical reversibility and cycling stability, making it more suitable for high-performance solid-state lithium battery applications.

**Figure 7:**
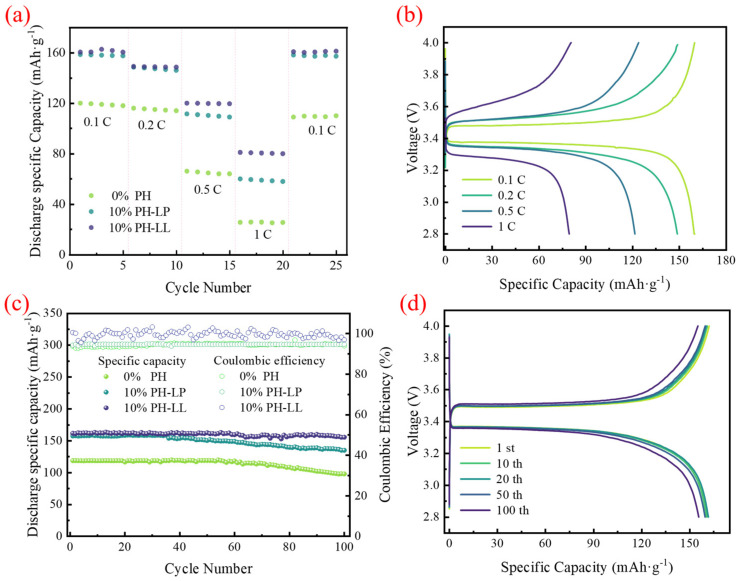
(**a**) Rate characteristics of LFP/GPE/Li batteries with 0% PH, 10% PH-LP, and 10% PH-LL electrolytes. (**b**) Charge–discharge behavior of LFP/Li batteries with 10% PH-LL electrolyte under different current rates (0.1–1 C). (**c**) Cycling performance of LFP/GPE/Li batteries with 0% PH, 10% PH-LP, and 10% PH-LL electrolytes. (**d**) Charge–discharge behavior of LFP/GPE/Li batteries with 10% PH-LL electrolyte at 0.1 C during selected cycles (1st, 10th, 20th, 50th, and 100th).

To examine the electrochemical behavior of 0% PH membrane, 10% PH-LP membrane and 10% PH-LL membrane, LFP/GPE/Li batteries were assembled in an argon-filled glovebox, and tested in a 25°C constant temperature box. Figure 7a shows the performance of 0% PH, 10% PH-LP, and 10% PH-LL batteries at various current rates ranging from 0.1 to 1 C. It is obvious that as the current increases, the discharge capacity of the batteries will decrease. At current rates between 0.1 and 0.5 C, the 0% PH cell exhibits initial discharge capacities of 120, 116, and 66 mAh·g^−1^. When the rate increases to 1 C, the capacity decreases significantly to only 25 mAh·g^−1^. Upon returning to 0.1 C, the discharge capacity recovers to 109 mAh·g^−1^, and the capacity recovers to merely 90% of its initial level. The specific capacity upon discharge of 10% PH-LP battery and 10% PH-LL battery re comparable at 0.1 and 0.2 C, whereas clear differences emerge at higher rates, with values of 115 vs. 120 mAh·g^−1^ at 0.5 C and 60 vs. 81 mAh·g^−1^ at 1 C. When the current returns to 0.1 C, both batteries rapidly regain their capacity. Figure 7b shows the charge–discharge curves of 10% PH-LL battery at different rates. The battery maintains a discharge plateau of 3.4 V, and the polarization voltage increases with the increase in the rate. At 0.1 C rate, the first discharge specific capacity of the battery is 160 mAh·g^−1^, reaching 94.1% of the theoretical specific capacity of LFP (170 mAh·g^−1^). It is worth noting that the performance of 10% PH-LL battery is better than that of the other two batteries under different discharge rates.

In addition to rate capability, long-term cycling stability is also crucial for evaluating the practical viability of lithium batteries. The cells were cycled at 0.1 C for 100 cycles within a cut-off voltage range of 2.8–4.0 V. Figure 7c shows the cycling stability of the three cells tested in a thermostatic chamber at 25 °C, while Figure 7d presents the charge–discharge profiles of the cell assembled with the 10% PH-LL membrane at the 1st, 10th, 20th, 50th, and 100th cycles. After 100 cycles, the 0% PH cell delivered a discharge specific capacity of 100 mAh·g^−1^, with a capacity retention of 83% and an average Coulombic efficiency of 93%. The relatively low Coulombic efficiency of the 0% PH cell indicates insufficient electrochemical reversibility, which may be associated with its lower ionic conductivity, larger polarization, and less stable electrode/electrolyte interface. Without ceramic fillers, the pristine PVDF-HFP-based membrane cannot effectively construct continuous Li^+^ transport pathways, leading to increased interfacial instability and possible side reactions during repeated cycling. In contrast, the 10% PH-LP and 10% PH-LL cells exhibited improved cycling stability. After 100 cycles, the 10% PH-LL cell maintained a higher discharge-specific capacity than the 10% PH-LP cell, with values of 155 and 135 mAh·g^−1^, respectively, and achieved an average Coulombic efficiency of approximately 99%. These results indicate that the incorporation of LLZTO not only provides more efficient Li^+^ transport pathways but also improves the interfacial compatibility among the polymer matrix, lithium salt, and inorganic filler, thereby suppressing interfacial degradation during cycling and significantly enhancing the cycling stability of the cell.

## 3. Conclusions

To summarize, a polymer-based gel electrolyte membrane was prepared using slit coating coupled with UV curing, and the structural and performance differences among filler-free, LATP-modified, and LLZTO-modified systems were systematically investigated. The incorporation of LLZTO markedly enhanced ionic transport in the electrolyte, achieving a room-temperature ionic conductivity of 3.4 × 10^−4^ S·cm^−1^, which was higher than those of 10% PH-LP (2.35 × 10^−4^ S·cm^−1^) and 0% PH (4.2 × 10^−5^ S·cm^−1^). Meanwhile, the interfacial impedance was significantly reduced. Microstructural analysis revealed that LLZTO was uniformly dispersed in the polymer matrix, effectively suppressing polymer crystallization and constructing continuous ion-conducting pathways, thereby producing a denser and more homogeneous electrolyte membrane. EDS results further verified the uniform elemental distribution, demonstrating excellent interfacial compatibility. In terms of Li^+^ transport characteristics, the Li^+^ transference number of 10% PH-LL reached 0.77, which was higher than that of 10% PH-LP (0.69), indicating that LLZTO provides stronger selectivity for Li^+^ migration. In addition, thermogravimetric analysis showed that this system exhibited a higher residual mass at elevated temperatures, confirming its improved thermal stability. Linear sweep voltammetry results indicated that the electrochemical stability window was broadened, suggesting its suitability for high-voltage battery systems. In terms of battery performance, the LiFePO_4_/GPEs/Li cell assembled with this electrolyte delivered a discharge specific capacity of 81 mAh·g^−1^ at 1 C, significantly outperforming 10% PH-LP (60 mAh·g^−1^) and 0% PH (25.6 mAh·g^−1^). After 100 cycles at 25 °C and 0.1 C, the capacity retention reached 96%, demonstrating excellent cycling stability. Benefiting from the advantages of slit coating, including controllable membrane thickness, continuous production, and scalability, the LLZTO-containing GPE exhibits superior ionic transport capability, reliable interfacial properties, and improved thermal robustness, providing a feasible material and process route for advanced high-energy lithium-ion batteries.

## 4. Materials and Methods

### 4.1. Materials

Poly(vinylidenefluoride) (PVDF, Mn = 600,000) and poly (vinylidenefluoride-co-hexafluoropropylene) (PVDF-HFP, Mn = 400,000) were supplied by Arkema (Colombes, France). Li_1.3_Al_0.3_Ti_1.7_P_3_O_12_ (LATP, D50 ≈ 0.6 μm) and Li_6.4_La_3_Zr1_.4_Ta_0.6_O_12_ (LLZTO, D50 ≈ 0.5 μm) were purchased from Shenzhen Kejing Zhida Technology Co., Ltd. Guangzhou, China). Lithium perchlorate (LiClO_4_, 99.99%) and N,N-Dimethylformamide (DMF, 99.8%) were obtained from Aladdin Reagent Co., Ltd. (Shanghai, China). The following materials were also obtained: *N*-methyl-2-pyrrolidone (NMP, ≥99.9%, Macklin Biochemical Technology Co., Ltd., Shanghai, China), Super-P conductive carbon black (≥99.5%, Kelude Chemical Technology (Shanghai) Co., Ltd., Shanghai, China), and lithium iron phosphate (LiFePO_4_, LFP, ≥99.5%, Dongguan Kelude New Energy Technology Co., Ltd., Guangzhou, China). The plasticizers used include ethoxylated trimethylolpropane triacrylate (ETPTA, Mn = 428, Qiyuan Pharmaceutical & Chemical Co., Ltd., Guangzhou, China), polyurethane acrylate (PUA, Degussa AG, Frankfurt, Germany), and 2-hydroxy-2-methyl-1-phenyl-1-propanone (HMPP, Shanghai Chem Technology Co., Ltd., Shanghai, China).

### 4.2. Preparation of LFP Cathode Sheet

Using NMP as the solvent, first, 3 drops of anhydrous NMP were mixed with 0.2 g of PVDF powder in a glass beaker. Through magnetic stirring, to the mixture was dissolved completely to form a homogeneous solution. Next, 1.6 g of LFP and 0.2 g of Super P carbon black were homogenized via manual grinding in an agate mortar for 40 min. After grinding, the obtained powder mixture was placed in a beaker, and a proper amount of NMP was introduced to facilitate uniform stirring. The beaker was covered with cling film and stirring was continued for 4 h to obtain a black cathode active slurry. Then, using a 50 μm scraper, the coating was applied, and the electrode sheet was placed in an 80 °C vacuum dryer for 36 h to completely remove the solvent. Finally, the as-prepared electrodes were punched into 14 mm discs and stored in an argon-filled glovebox (H_2_O/O_2_ < 0.01 ppm) before further cell assembly.

### 4.3. Preparation Process of Gel Polymer Electrolyte Membranes (GPEs) via the Slit-Coating Process

**Figure 8:**
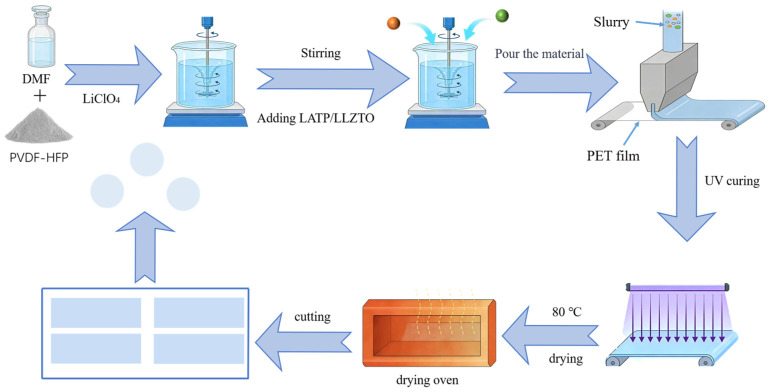
The process of preparing the membrane.

First, 30 g of PVDF-HFP was dissolved in 200 g of DMF, and a homogeneous polymer solution was obtained under ambient conditions using magnetic stirring. Subsequently, LiClO_4_ and LATP or LLZTO fillers with different contents were introduced into the system, followed by continuous stirring for 6 h to obtain a homogeneous and stable composite slurry. The filler contents were controlled at 0, 5, 10, and 15 wt% relative to the mass of PVDF-HFP. Here, 0% PH refers to the filler-free PVDF-HFP-based GPE membrane without LATP or LLZTO addition. Afterward, appropriate amounts of ETPTA and PUA were added as UV-curable monomers, and HMPP was introduced as the photoinitiator to construct a UV-curable precursor system. The slurry was then coated onto a PET release film substrate using the slit-coating process. By adjusting the gap of the slit-die head, the coating thickness was controlled, and the prepared slurry was continuously delivered to the slit-die head and uniformly extruded to form a continuous wet film on the PET release film. The coated wet film was first subjected to in situ photopolymerization under ultraviolet irradiation to rapidly construct an initial crosslinked network and improve the structural stability of the membrane. Subsequently, the cured membrane, together with the substrate, was placed in a drying oven, where the solvent was further removed under heating conditions to obtain a dense and uniform gel polymer electrolyte membrane. According to the type and content of the inorganic filler, the prepared membranes were labeled as 0% PH, 5% PH-LP, 10% PH-LP, 15% PH-LP, 5% PH-LL, 10% PH-LL, and 15% PH-LL. Finally, the as-prepared GPEs were punched into 19 mm discs and stored in an argon-filled glovebox with H_2_O/O_2_ contents below 0.01 ppm for subsequent cell assembly and electrochemical evaluation. The preparation process is shown in Figure 8.

### 4.4. Assembly of LFP/GPE/Li Cells

**Figure 9:**
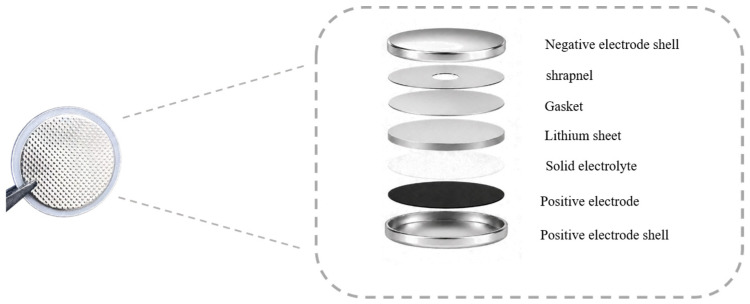
Schematic illustration of the assembly process and internal configuration of the CR2025 LFP/GPE/Li coin cell.

The LFP cell was assembled into a CR2025 coin cell using the cathode, solid-state electrolyte membrane, and lithium metal foil. The assembly process of the CR2025 cell is shown in Figure 9. In an argon-filled glovebox, the cell components were stacked in the following order: positive case, cathode, solid-state electrolyte membrane, lithium metal foil, spacer, spring, and negative case. After assembly, the cells were rested in the glovebox for 8–10 h before electrochemical testing.

### 4.5. Materials Characterization

The crystal structure of the GPE samples was analyzed by XRD (DX-2700, Dandong, China) with Cu Kα radiation operated at 40 kV and 30 mA. Surface and fracture morphologies were observed using SEM (Phenom Spectra G2, Shanghai, China), where cross-sections were obtained by cryogenic fracture in liquid nitrogen and sputter-coated with gold prior to imaging. Mechanical properties were determined on a universal testing system (WDW-5, Tenson, Jinan, China). Thermal behavior was evaluated by thermogravimetric analysis (Netzsch F3 Tarsus, METTLER TOLEDO, Germany) under a nitrogen atmosphere from 30 to 800 °C at a heating rate of 10 °C·min^−1^ with a gas flow of 100 mL·min^−1^. Functional groups were identified by FTIR spectroscopy (Spectrum 100, PerkinElmer, Shelton, DC, USA) within the wavenumber range of 500–4000 cm^−1^.

### 4.6. Electrochemical Performance of GPE Membranes

Electrochemical impedance spectroscopy (EIS) measurements were performed using SS/GPEs/SS symmetric cells to evaluate the electrochemical properties of the GPEs. The tests were carried out on an electrochemical workstation (DH 7000, Jiangsu, China) at room temperature under open-circuit conditions, with a frequency range from 0.1 Hz to 1 MHz and an AC amplitude of 10 mV. The obtained impedance data were used to assess the suitability of the GPEs for solid-state battery applications. The ionic conductivity (σ) was subsequently calculated according to the following equation.

(1) $$\begin{array}{c} \sigma =\frac{L}{RS} \end{array}$$

Here, *σ* is the ionic conductivity, *L* is the thickness of the electrolyte membrane, *R* is the bulk resistance obtained from EIS fitting, and *S* is the effective contact area between the stainless-steel electrode and the electrolyte membrane, which was 2.0096 cm^2^ in this work.

The lithium ion migration number (*t_Li_*^+^) is used to evaluate the ability of a membrane to transfer lithium ions [53]. The lithium-ion transference number (*t_Li_*^+^) of the GPEs was determined using Li/GPE/Li symmetric cells by combining electrochemical impedance spectroscopy (EIS) with DC polarization measurements on an electrochemical workstation. Firstly, the initial impedance value (*R*_0_) was obtained through EIS testing. Then, a polarization voltage of 0.05 V was applied and maintained for 4000 s. The initial current value (*I*_0_) and the steady-state current value (*Is*) were measured respectively. After removing the polarization voltage, the impedance spectrum was measured again, and finally the polarization-induced interfacial resistance (*Rs*) was obtained. The value of the lithium-ion transference number (*t_Li_*^+^) was determined using the equation given below.

(2) $$\begin{array}{c} t_{{Li}^{+}}=\frac{I_{S}\left(\Delta V-R_{0}I_{0}\right)}{I_{0}\left(\Delta V-R_{S}I_{S}\right)} \end{array}$$

Here, *I*_0_ and *I_S_* represent the initial and steady-state currents in the current-time curve, respectively; *R*_0_ and *R_S_* represent the initial and steady-state resistances obtained from the AC impedance curve, respectively; and *ΔV* represents the applied polarization voltage (0.05 V).

To evaluate the stability window of the GPEs, Li/GPE/SS cells were assembled and examined via a linear potential sweep using an electrochemical system. The measurements were performed over a voltage range of 2.0–6.0 V at a scan rate of 5 mV·s^−1^, and the obtained results were used to determine the stability limits of the GPEs.

To investigate the stability behavior and redox reversibility of GPEs with different inorganic fillers, LFP/GPEs/Li cells were assembled and analyzed by cyclic voltammetry using an electrochemical system. The measurements were conducted within a voltage range of 2.8–4.0 V at a scan rate of 0. 2 mV·s^−1^.

The rate capability and cycling behavior of the cells were evaluated using a battery testing system (Neware, Dongguan, China). Cell fabrication was conducted in an argon-filled glovebox, employing LiFePO_4_ as the cathode and lithium metal as the anode, with the prepared GPEs serving as the electrolyte layer. CR 2025-type coin cells were assembled and tested within a voltage range of 2.8–4.0 V. The discharge capacity of the battery under different rate conditions and the performance stability during the long cycle process were mainly evaluated.

## Figures and Tables

**Table 1:** Comparison of electrochemical performance of PVDF-HFP-based polymer electrolytes with different inorganic fillers.

Polymer Matrix	Filler	Filler Content	*σ* (S·cm^−1^)	*t_Li_^+^*	LSV	Cell Configuration	Reference
PVDF-HFP	LATP	10 wt%	2.35 × 10^−4^	0.69	~5.4 V	LFP/GPE/Li	This work
PVDF-HFP	LLZTO	10 wt%	3.40 × 10^−4^	0.77	~5.6 V	LFP/GPE/Li	This work
PEO/PVDF-HFP	LLZTO	-	2.65 × 10^−4^	0.47	5.55 V	LiFePO_4_/CSPE/Li	[46]
PVDF-HFP-based GPE	TiO_2−x_ nanofibers	-	1.90 × 10^−4^	0.70	5.50 V	Li/LiFePO_4_ and Li/NCM811	[47]

**Table 2:** Experimental measurement parameters of solid electrolytes and the calculated lithium ion mobility (*t_Li_^+^*) at room temperature.

Solid Polymer Electrolyte	*I*_0_ (mA)	*I_S_* (mA)	*R*_0_ (Ω)	*R_S_* (Ω)	*t_Li_* ^+^
10% PH-LP	0.01915	0.014	229.809	227.36	0.69
10% PH-LL	0.0325	0.02644	154.893	150.188	0.77

## Data Availability

The data are contained within the article.
